# Membrana de cáscara de huevo para la curación de heridas superficiales en ratones

**DOI:** 10.7705/biomedica.6192

**Published:** 2022-06-01

**Authors:** Noelia Mendoza, Georgina Chávez, Omar Araya

**Affiliations:** 1 Carrera de Ingeniería Biomédica, Facultad de Ingeniería, Universidad Católica Boliviana “San Pablo”, La Paz, Bolivia Universidad Católica Boliviana San Pablo Facultad de Ingeniería Universidad Católica Boliviana “San Pablo” La Paz Bolivia

**Keywords:** cicatrización de heridas, heridas y lesiones, ovi gallinae pellicula, ratones, terapéutica, Wound healing, wounds and injuries, *ovi gallinae pellicular*, mice, therapeutics

## Abstract

**Introducción.:**

Las membranas de la cáscara de huevo presentan propiedades beneficiosas para la regeneración de tejidos y sus aplicaciones biomédicas son importantes.

**Objetivo.:**

Demostrar la efectividad de las membranas de la cáscara de huevo no fecundado de gallina en el tratamiento de heridas abiertas superficiales en ratones, en comparación con el procedimiento convencional.

**Materiales y métodos.:**

Se hizo una herida superficial lineal de 15 mm en la espalda de 10 ratones albinos machos. Los ratones se dividieron en cuatro grupos, uno no recibió ningún tratamiento y los otros tres sí: uno, tratamiento convencional, otro, con membranas de huevo directamente aplicadas a la herida y, el otro, con membranas en forma de polvo.

La evolución de las heridas se registró en fotografías y se calculó la tasa de reducción de la longitud de la herida, así como el tiempo y el porcentaje de curación. Los porcentajes de curación se analizaron con ANOVA y la prueba de Dunnett (p<0,05).

**Resultados.:**

Con los tratamientos con membranas de huevo y polvo de membrana, se logró una tasa de reducción de longitud de 1.009 y 1.020 mm/día, respectivamente, y un tiempo de curación de 12 días, en tanto que, con el tratamiento convencional, la tasa de reducción fue de 0,852 mm/día y la curación se dio en 16 días. El análisis estadístico mostró diferencias significativas entre los tratamientos con membrana de huevo y el tratamiento convencional.

**Conclusiones.:**

Las membranas de la cáscara de huevo aplicadas de forma directa y en polvo resultaron más efectivas que la aplicación del procedimiento convencional en el tratamiento de heridas abiertas superficiales en ratones.

## Abstract (en)

**Introduction:**

Eggshell membranes offer beneficial properties for tissue regeneration and their biomedical applications are important.

**Objective:**

To demonstrate the effectiveness of hen unfertilized eggshell membranes in the treatment of superficial open wounds in mice compared to the conventional procedure.

**Materials and methods:**

15-mm linear wounds were made on the back of 10 male CF-1 mice. The mice were divided into four groups. One group did not receive any treatment and the other three were given conventional treatment, eggshell membranes dressing, and eggshell membranes powder. The evolution of the wounds was registered by photographs.

We measured wounds length and healing time and calculated the length reduction rate and the percentage of healing. The healing percentages were analyzed by ANOVA with a post hoc Dunnett test (p<0.05).

**Results:**

The treatments with eggshell membranes and membrane powder had a wound- length reduction rate of 1.009 and 1.020 mm/day, greater than the conventional treatment, of 0.852 mm/day, and a healing time of 12 days, i.e., less than the 16-day conventional treatment. The statistical analysis showed significant differences between eggshell membrane treatments and the conventional treatment.

**Conclusions:**

Eggshell membrane dressing and eggshell membrane powder applied to open superficial wounds in mice were more effective than the conventional treatment in regenerating injured tissue in mice.

La piel es uno de los órganos más grandes del organismo. Además de cubrir la superficie del cuerpo, cumple funciones esenciales en la inmunidad del organismo, su termorregulación y su protección ^1^. Al estar en contacto constante con el exterior, tiende a sufrir lesiones, más frecuentemente heridas superficiales abiertas ^2^.

Las heridas, entendidas como el resultado de una ruptura en la continuidad de la piel ^3^, pueden implicar riesgo para la vida del paciente dependiendo de su gravedad. Por ello, es vital atenderlas en el menor tiempo posible, es decir, limpiarlas y desinfectarlas rigurosamente ^4^. El método convencional para tratar las heridas implica el empleo de antisépticos, tiras emplásticas y apósitos que buscan una rápida cicatrización, y la restauración de la superficie afectada ^4^. Sin embargo, en muchos casos es inevitable que ciertas heridas tarden en cicatrizar y terminen en una infección, a pesar de su correcto manejo, la cual hace necesario un tratamiento más agresivo y complicado, que resulta en una cicatrización menos estética y afecta tanto al paciente como al personal de salud que lo atiende ^5^. Por ello, se buscan nuevas alternativas para tratar las heridas que sean menos agresivas y dolorosas para el paciente, le ahorren tiempo y esfuerzo al personal de salud e impliquen un menor gasto de recursos.

Las membranas de la cáscara de huevo tienen una estructura fibrosa ^6^ compuesta por una red de biopolímeros ^7^, y se encuentran entre la cáscara y el albumen del huevo. Están constituidas por proteínas en el 80 al 85 % y el 10 % corresponde al colágeno ^6^, el cual puede clasificarse como colágeno de tipo I ^8^, aunque también están el de tipo V y el de tipo X ^6^. Se han reportado también otras proteínas específicas de las membranas, como las reguladoras del complemento asociado con la membrana (*Complement Regulatory Protein*, CREM o antes Cremp) ^9^, cuya principal función es la protección contra el ataque de patógenos ^10^.

Chato, *et al.,* demostraron mediante microscopía óptica que las membranas de la cáscara de huevo promueven el crecimiento de fibroblastos en los humanos, lo que favorece la cicatrización ^11^. En otros estudios, se halló que las membranas de la cáscara de huevo presentan propiedades que las convierten en candidatas para aplicarlas a manera de vendajes, injertos de piel o productos de reemplazo de tejidos y contribuir, así, a la regeneración de tejidos dañados (Solé MA. Valorización de residuos agroindustriales. Primer Simposio Regional La Bioeconomía y el Territorio Inteligente. Cuyo, Argentina; 2016) ^12^^-^^15^. No obstante, según la Guía de Educación Ambiental en Gestión de Residuos Sólidos de Bolivia ^16^, la cáscara de huevo se considera un residuo sólido orgánico, por lo que comúnmente se deposita en los vertederos sin ningún tratamiento previo.

Según el Boletín Estadístico Anual de la Asociación de Avicultores (ADA) del departamento de Cochabamba, Bolivia ^17^, la producción de huevo a nivel nacional para el 2017 llegó a 1.882,73 millones de unidades. Un huevo de tamaño promedio pesa alrededor de 57 g y la parte desechable (cáscara) constituye el 11 % ^18^. Por lo tanto, se calcula que en el país se producen, aproximadamente, 11.804,72 t de residuos de cáscara de huevo al año. En La Paz, los principales programas de aprovechamiento de residuos sólidos orgánicos están destinados a la actividad de compostaje y lumbricultura ^19^, pero podrían aprovecharse en otros campos, especialmente en la medicina.

En este contexto, el objetivo del presente trabajo fue demostrar la efectividad de las membranas de la cáscara de huevo en el tratamiento de heridas abiertas superficiales en ratones, aplicadas directamente sobre la lesión o en forma de polvo, como una alternativa efectiva y económica.

## Materiales y métodos

### Diseño y población de estudio

El presente es un estudio de tipo descriptivo experimental, con un diseño de preprueba, posprueba y grupo de control ^20^. Se adquirieron 10 ratones albinos machos de mediana edad de la cepa CF1 en el Bioterio del Instituto Nacional de Laboratorios de Salud (INLASA) “Néstor Morales Villazón” y se dividieron en cuatro grupos: un grupo de control y tres grupos experimentales expuestos cada uno a una modalidad de tratamiento. El grupo de control incluía un individuo y, los grupos experimentales, tres cada uno, todos distribuidos al azar. Antes de iniciar la etapa experimental, los ratones tuvieron un tiempo de adaptación al nuevo ambiente y a la manipulación del investigador.

El estudio incluyó las etapas de preparación del ambiente experimental, preparación del material para los tratamientos, creación y registro fotográfico de las heridas, aplicación de los tratamientos y seguimiento de la evolución de las heridas y, por último, el procesamiento y análisis de los datos obtenidos.

El macroambiente y el microambiente en que se mantuvieron los ratones se ajustaron a las recomendaciones de la “Guía de manejo y cuidado de animales de laboratorio” del Instituto Nacional de Salud de Lima ^21^. Se prepararon jaulas individuales para cada ratón, con acceso libre a comida y agua, y exposición a 12 horas de oscuridad y 12 horas de luz.

### Procedimiento experimental

Cumplido el mes de adaptación de los ratones, se inició la etapa de experimentación. Se rasuró un área aproximada de 20 mm^2^ en la espalda de los ratones y se aplicó un anestésico tópico (lidocaína al 5 %) en ella. Se hizo una herida superficial lineal, aproximadamente, de 15 mm de longitud en la parte inferior central de la espalda de cada uno de los ratones en un mismo día y cumpliendo un horario específico que se inició con el grupo control, prosiguió con el grupo experimental 1, luego, el grupo experimental 2 y, finalmente, el grupo experimental 3. Cada herida se registró mediante una fotografía tomada con un celular Samsung J4, que contaba con una cámara de 13 megapíxeles ubicada a una altura y posición fijas con respecto al área de experimentación.

### Preparación y aplicación de tratamientos

Se aplicaron los tratamientos y se hizo el registro fotográfico cada tres días durante 15 días hasta el cierre total de la lesión, es decir, un total de cinco registros por ratón. En el primer grupo experimental, se aplicó el tratamiento convencional con agua oxigenada de 10 vol. En el segundo grupo experimental, se empleó el tratamiento con membranas de cáscara de huevo previamente retiradas de un huevo desinfectado con alcohol (70 %) con la ayuda de una pinza desinfectada y directamente aplicadas a la herida; se utilizó un segmento con las dimensiones adecuadas para cubrir toda la herida de cada uno de los tres ratones de este grupo. El tercer grupo experimental fue tratado con polvo de membrana, cubriendo totalmente la herida.

El material se preparó con las membranas de la cáscara de cinco huevos almacenadas en un recipiente de plástico sin tapa que se guardó en un lugar seco y limpio hasta que las membranas se desecaron a temperatura ambiente. Una vez se secaron completamente, se pulverizaron en un mortero de vidrio pequeño y el polvo resultante se almacenó en el recipiente de plástico hasta su utilización. El grupo de control no recibió ningún tipo de tratamiento. Finalizada la etapa de experimentación, se prosiguió con el procesamiento de los datos recolectados.

### Variables y mediciones

Se determinaron, como variable independiente, el tratamiento aplicado y, como variable dependiente, la herida. La manipulación de la variable independiente fue de bajo grado (presencia y ausencia), y se combinó con las tres modalidades de tratamiento: el convencional, otro con membranas de cáscara de huevo directamente aplicadas a la herida y otro con las membranas en forma de polvo.

Para la medición del efecto de los tratamientos, se calcularon la variación y la tasa de reducción de la longitud de la herida, el porcentaje de curación y el tiempo de curación. La longitud de la herida se determinó con base en el análisis de las fotografías y la ayuda del programa ImageJ empleado en otros estudios sobre la evolución de heridas ^22^. Para garantizar la exactitud y la precisión de las mediciones, se tomaron 20 datos de longitud por imagen que luego se utilizaron para calcular la media del porcentaje de curación y su correspondiente desviación estándar.

La tasa de reducción de la longitud de la herida se calculó con la fórmula presentada en Siavash, *et al.*^23^:




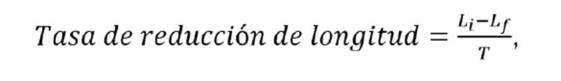




donde L_i_ es la longitud inicial de la herida y L_f_ es su longitud al final del estudio (expresadas en mm), y T es la duración del estudio expresada en días.

Para el porcentaje de curación, se usó la fórmula adaptada para la medición de longitud (mm) presentada en el estudio de Jessup ^24^.




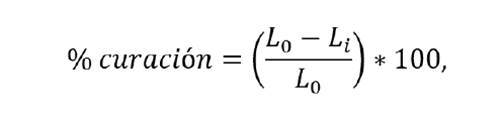




donde L_0_ es la longitud inicial de la herida, L_i_ es la longitud en la medición i, e i es el número de la medición.

El tiempo de curación se calculó desde el primer día de experimentación hasta la cicatrización total de cada herida.

### Análisis estadístico

Se utilizaron un análisis de varianza (ANOVA) y el test de Dunnett para comparar cada grupo experimental con el grupo control ^25^. Con el ANOVA se determinó si existía una diferencia significativa entre los porcentajes de curación de todos los grupos de estudio, incluido el grupo control. El análisis se hizo en los días 4, 7, 11 y 15. Si el valor de significación era mayor de 0,05, no se hacía el test de Dunnett, pues ello significaba que la media de los porcentajes de curación de todos los grupos no presentaba una diferencia significativa. Si el valor de significación resultaba menor o igual a 0,05, entonces se hacía el test de Dunnett para evaluar cuál de los tratamientos presentaba una diferencia significativa con respecto al grupo de control.

### Consideraciones éticas

El estudio fue aprobado por las autoridades de la Universidad Católica Boliviana “San Pablo” y el procedimiento de cuidado y manejo de los animales fue avalado por su Comité de Ética, en conformidad con las directrices sugeridas por las guías del *Canadian Council on Animal Care* (CCAC) para los procedimientos experimentales con ratones ^26^.

## Resultados

En el cuadro 1 se presentan los valores promedio de la longitud de las heridas medida cada tres días durante los 15 días del estudio. Los valores de los grupos experimentales (de tres individuos cada uno) corresponden al promedio y la desviación estándar de todos sus individuos.

**Cuadro 1:** Longitud de herida en mm de ratones albinos en los tres grupos de experimentación y el grupo de control a los 0, 4, 7, 11 y 15 días de estudio

	Grupo								
	Día	Control		1er.	EXP	2do.	EXP	3er.	EXP
		M	DE	M	DE	M	DE	M	DE
Longitud (mm)	0	15,5	0,23	15,5	0,56	15,1	0,07	15,3	0,47
	4	14,6	0,21	14,4	0,24	14,6	0,15	14,2	0,47
	7	11,5	0,03	11,6	0,49	9,8	1,03	12,0	1,95
	11	10,8	0,04	10,5	0,30	3,4	0,64	8,1	3,90
	15	8,4	0,01	8,0	0,06	*	*	*	*

M: media; DE: desviación estándar

* cicatrización completa

A los 15 días de haberse producido la herida, los individuos A1 y A3 del primer grupo experimental (tratamiento convencional) presentaron cicatrización completa, lo que implicó que la longitud de la herida ya no pudo ser medida. En el segundo grupo experimental (con membrana de huevo directamente aplicada sobre la lesión), el individuo B1 constituyó un caso extraordinario, pues presentó una cicatrización completa de la herida a los siete días. Las heridas de los individuos B2 y B3 cicatrizaron completamente a los 15 días. En el tercer grupo experimental 3 (con polvo de membrana de huevo), los individuos C1 y C2 presentaron cicatrización completa a los 15 días. El individuo C3, otro caso extraordinario, presentó cicatrización completa a los siete días. En el individuo de control no se alcanzó la cicatrización completa durante el periodo de experimentación.

Para observar el efecto de los tratamientos sobre la longitud de la herida, se calculó la tasa de reducción de la longitud (cuadro 2). Se registró, además, el tiempo promedio de curación de cada grupo a partir del día en que se hizo la herida hasta su cicatrización completa, en cada individuo dentro de cada grupo. En el segundo y el tercer grupo (tratados con membrana de huevo), la tasa de reducción de la longitud fue mayor que en el grupo que no recibió tratamiento y que en el primer grupo (tratamiento convencional con agua oxigenada) El tiempo de curación fue mayor en el grupo control y el primer grupo, en tanto que, en los grupos tratados con membranas de huevo, este fue menor.

**Cuadro 2:** Tasa de reducción de la longitud de la herida y tiempo medio de curación en los tres grupos de experimentación y en el de control

Grupo	Tasa promedio reducción de longitud (mm/día)	Tiempo medio de curación (días)
Control	0,476	18
1er. EXP	0,852	16
2do. EXP	1.009	12
3er. EXP	1.020	12

La tasa de reducción de la longitud de la herida de los tratamientos con membranas de huevo directamente aplicadas a la lesión (1.009 mm/día) y con polvo de membrana (1.020 mm/día) fue mayor que la del grupo que no recibió tratamiento (0,476 mm/día) y que la del tratamiento convencional (0,852 mm/ día). Puede concluirse, entonces, que el tratamiento con membranas de huevo tuvo un efecto positivo en la reducción de la longitud de estas heridas abiertas superficiales, en comparación con el tratamiento convencional.

El tiempo de curación promedio con las membranas de huevo y con el polvo de membrana, ambos de 12 días, fue menor que el del grupo que no recibió tratamiento (18 días) y que el de tratamiento convencional (16 días). Es decir, el tratamiento con membranas de huevo en sus dos formas tuvo un efecto positivo en la aceleración de la curación de las heridas, en comparación con el tratamiento convencional. En el cuadro 3 se resumen y comparan los porcentajes del grupo de control y de los tres grupos experimentales. Puede notarse que solo los grupos experimentales 2 y 3 alcanzaron un porcentaje de curación del 100 % en los 15 días de estudio.

**Cuadro 3:** Comparación de porcentajes de curación en ratones albinos de los tres grupos de experimentación y el de control a los 0, 4, 7, 11 y 15 días de estudio

		Grupo				Anova	
Día		Control	1er. EXP	2do. EXP	3er. EXP	F	P
% curación	0	0	0	0	0	-	-
	4	5,8	7,0	3,6	7,4	0,72	0,568
	7	26,2	24,9	56,9	46,7	0,818	0,519
	11	30,5	32,1	85,0**	64,1	5,582	0,023
	15	46,0	83,4	100**	100**	9,375	0,005

* Diferencias significativas entre los grupos, incluido el control (p≤0,05)

** Diferencias significativas entre grupos experimentales y grupo de control (prueba de Dunnet: p≤0,05)

Los resultados del test de Dunnett en el día 11 indicaron que el tratamiento con membranas directamente aplicadas a la herida (segundo grupo) presentaba una diferencia significativa con respecto al grupo de control (p=0,011). Los resultados en el día 15 indicaron que los tres tratamientos, grupo 1 (p=0,016), grupo 2 y grupo 3 (p=0,002), fueron efectivos con respecto al grupo de control. Sin embargo, al comparar los resultados de las membranas con el tratamiento convencional, pudo determinarse que los tratamientos con membranas tuvieron una diferencia más significativa con respecto al otro.

**Figura 1:**
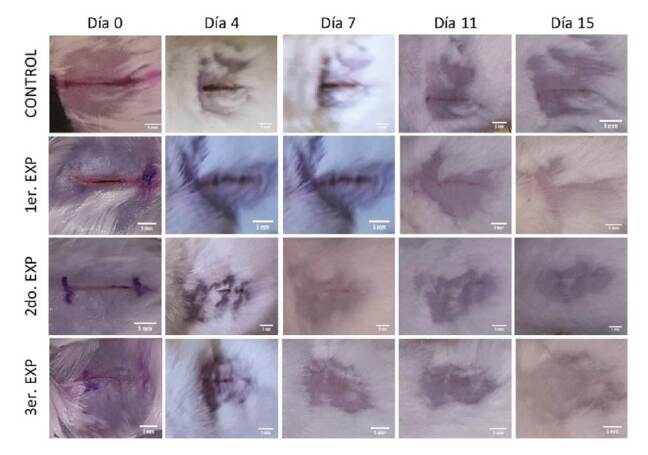
Progreso de la curación de la herida en ratones tratados con polvo de membrana de huevo (3er. EXP), membrana de huevo directamente aplicada en la herida (2do. EXP), tratamiento convencional (1er. EXP) y sin tratamiento a lo largo de 15 días

Con base en los resultados del análisis estadístico, se determinó que los tratamientos con membrana de huevo aplicada en sus dos formas: directamente sobre la lesión y en forma de polvo, presentaron un efecto más positivo en la curación de heridas abiertas superficiales que el tratamiento convencional (p≤0,05). En la figura 1, se reflejan el progreso de la curación de la herida en un individuo de cada grupo de estudio mediante las fotografías registradas a lo largo de los 15 días de experimentación y el contraste de los grupos experimentales con el grupo de control.

## Discusión

La membrana de huevo se ha usado tradicionalmente como vendaje natural para el tratamiento de cortaduras y quemaduras desde hace más de 400 años ^13^. Su composición química rica en glucoproteínas, colágeno y carbohidratos como el ácido hialurónico ^13^, su adherencia debida a la estructura de red, su capacidad de retención de humedad, su permeabilidad ^27^, su propiedad de acelerar la epitelización de heridas y aliviar el dolor ^14^, entre otras características, hacen de ella un prometedor biomaterial para aplicarlo como apósito en el tratamiento de heridas.

En este estudio, se evaluó el uso de las membranas de huevo aplicadas de dos formas, directamente o en polvo, para promover la cicatrización de heridas superficiales por incisión longitudinal en ratón ^28^. Se recurrió a este modelo porque este tipo de herida es más corriente en la vida cotidiana que las heridas por escisión ^2^^,^^28^. Sin embargo, el hecho de que las heridas en los roedores sanen por contracción representa una limitación al usarlos como modelos de heridas humanas ^29^, por lo que suelen usarse estrategias para evitar dicha contracción, tales como el uso de férulas en el caso de escisiones ^9^, o la costura de los bordes de la herida con hilo quirúrgico en el caso de incisiones ^28^^).^ Estas estrategias no se consideraron en nuestro estudio por tratarse de heridas superficiales, por lo que los resultados obtenidos podrían presentar variaciones al aplicarse en heridas humanas.

Los resultados del presente estudio demostraron la efectividad de las membranas de cáscara de huevo en el tratamiento de heridas abiertas superficiales en ratones mediante el estudio macroscópico de la cicatrización y complementan aquellos a nivel microscópico, como los de Vuong, *et al.*, quienes demostraron que la membrana de huevo en polvo posee propiedades inmunomoduladoras y antinflamatorias, además de mejorar la proliferación de fibroblastos y queratinocitos, importantes en la primera fase de cicatrización de las heridas ^13^^,^^30^. Nuestros resultados sugieren que la membrana de huevo en polvo puede emplearse como un producto de bajo costo para el tratamiento de heridas superficiales.

Además, el análisis estadístico del día 11 de experimentación sugiere que el tratamiento con membrana de huevo directamente aplicada en la herida tiene un mejor efecto sobre el porcentaje de curación que los otros dos tratamientos analizados en este estudio, lo que coincide con lo hallado por Guarderas, *et al.*, quienes señalan que la aplicación directa (sin procesar o moler) de la membrana como apósito sobre las heridas mejora significativamente su cicatrización ^12^.

El uso del polvo de membrana de huevo de gallina no fecundada en la elaboración de productos para la curación de heridas implicaría una reducción en la producción de residuos sólidos orgánicos depositados en los vertederos nacionales y tendría un impacto ambiental positivo, ya que, eliminados en grandes cantidades, estos residuos pueden generar lixiviados y gases de efecto invernadero al descomponerse, que son 21 veces más dañinos para el ambiente que el CO_2_^31^.

El uso de la membrana de huevo como materia prima en productos con acción cicatrizante o efecto dermatológico refuerza los resultados obtenidos aquí en torno a sus propiedades en el tratamiento de heridas superficiales cutáneas. Por ejemplo, se desarrolló una fibra adherente a base de membrana de huevo que puede usarse como apósito para heridas ^27^. Por otra parte, Ballester, *et al.*, desarrollaron un producto cicatrizante compuesto por membranas de huevo que puede aplicarse como estimulante del crecimiento de tejido y que permite, además, acortar el tiempo de cicatrización ^32^.

Se concluye que las membranas de huevo aplicadas de forma directa o en polvo sobre heridas abiertas superficiales en ratones, fueron significativamente efectivas ya en el día 11 (p<0,05) y permitieron el cierre completo de la herida en un tiempo promedio de 12 días con la aplicación del tratamiento cada tercer día. La aplicación de membrana de la cáscara de huevo representa una alternativa terapéutica en este tipo de heridas y mejora la calidad de la atención de los pacientes, pues acelera el proceso de curación y es menos agresiva y dolorosa.
